# Complex Transfusion Management in a Sickle Cell Patient With Anti-Fy3 Alloimmunization: A Case Report

**DOI:** 10.7759/cureus.60939

**Published:** 2024-05-23

**Authors:** Karen Claesen, Bert Heyrman, Pieter De Schouwer, Sarah Mahieu

**Affiliations:** 1 Laboratory for Clinical Biology, Ziekenhuis Netwerk Antwerpen (ZNA), Antwerp, BEL; 2 Department of Haematology, Ziekenhuis Netwerk Antwerpen (ZNA), Antwerp, BEL

**Keywords:** blood group, transfusion medicine, adult sickle cell anemia, hematology laboratory, blood supply management, anti-fy3 antibodies

## Abstract

Fy3 is a high-prevalence red blood cell antigen of the Duffy (Fy) blood group system. Anti-Fy3 antibodies are rare and solely arise in individuals with a Duffy null phenotype (Fy(a-b-)), which is a phenotype that mainly occurs in people of African descent. Clinically, anti-Fy3 antibodies can cause both acute and delayed hemolytic transfusion reactions in adults as well as hemolytic disease in fetuses and newborns. Here, we report the case of a 26-year-old male with sickle cell disease (SCD) and a history of anti-E alloantibodies, who was admitted to the hospital with a vaso-occlusive crisis (VOC) and associated low hemoglobin (Hb) level. For the latter he received one unit of antigen-matched and crossmatch-compatible packed red blood cells (pRBCs) without complications. Ten days later the patient was readmitted with a further VOC and associated low Hb level, again requiring a red cell transfusion. However, no crossmatch-compatible pRBCs could be identified. Laboratory testing demonstrated pan-reactivity with additional reference testing demonstrating the presence of anti-E, anti-Fy3 and anti-Jk^b^ alloantibodies. This case highlights the diagnostic and therapeutic challenges associated with blood transfusion in SCD patients with rare alloimmunization profiles.

## Introduction

Sickle cell disease (SCD) is an inherited hemoglobin (Hb) disorder characterized by the presence of hemoglobin S (HbS), which causes red blood cells (RBC) to become rigid and sickle-shaped [1,2]. Clinically, these RBC abnormalities manifest in chronic hemolytic anemia and cycles of microvascular occlusion leading to end-organ ischemia-reperfusion injury and infarction [2,3].

Blood transfusions play a vital role in managing acute and chronic complications of SCD by ameliorating anemia, preventing and treating complications, and improving overall quality of life. However, undergoing multiple transfusions carries a risk of alloimmunization [4,5]. This not only increases the risk of hemolytic transfusion reactions, but also significantly complicates the selection of compatible blood products for future transfusions [6].

The Duffy (Fy) blood group system, named after a man with hemophilia who developed a novel antibody after multiple blood transfusions, is primarily characterized by the antigens Fy^a^ and Fy^b^. Four main Duffy phenotypes can be distinguished: Fy(a+b+), Fy(a+b-), Fy(a-b+) and Fy(a-b-). Additional high-prevalence Fy antigens include Fy3 and Fy6. Individuals with the Fy(a-b-) phenotype lack both Fy3 and Fy6 antigen expression. Fy antigens are located on the Duffy glycoprotein or Duffy antigen receptor for chemokines (DARC), and are expressed not only on erythrocytes but also on other cell types throughout the body. Antibodies to Fy antigens are usually clinically significant and have been reported to cause hemolytic disease of the fetus and newborn [7-9].

Here we describe the case of a patient with SCD, in whom the emergence of anti-Fy3 together with anti-E- and anti-Jk^b^ alloantibodies greatly complicated transfusion management during a vaso-occlusive sickle cell crisis (VOC).

## Case presentation

A 26-year-old male patient with homozygous SCD (HbSS) presented at the emergency department with symptoms of fever, cough, back pain and pain over the right lower quadrant of the abdomen since one week, not amenable to analgesics. The patient had a history of recurrent VOCs and received multiple blood transfusions over the years, which resulted in the formation of anti-E alloantibodies and auto-antibodies with broad specificity. In 2012, the patient’s blood group antigen profile was determined as O positive, C- E- c+ e+ K- k+ Fy(a-b+) Jk(a+b-) M+ N- S- s+ by molecular testing. Upon admission, the patient’s vital signs were stable and general physical examination was significant for tenderness in the right subcostal area. Initial laboratory investigations indicated normocytic normochromic anemia and acute hemolysis (Table 1), suggestive of an acute VOC. Hemoglobin electrophoresis showed 86.7% HbS, 3.5% HbA2 and 9.8% HbF. 

**Table 1:** Laboratory parameters of the patient during hospital admission LDH: lactate dehydrogenase

	Initial presentation				Re-presentation						Reference ranges
	Day 1	Day 2	Day 3	Day 4	Day 1	Day 3	Day 4	Day 5	Day 6	Day 8	
Hemoglobin (g/dL)	8.0	7.0	7.8	7.4	7.4	6.5	5.9	6.7	6.9	6.6	12.9 - 16.4
LDH (U/L)	502	473	521	440	525	811	961	-	-	412	135 - 225
Total bilirubin (mg/dL)	3.0	-	3.4	3.1	2.2	3.1	3.8	-	1.7	-	< 1.2
Indirect bilirubin (mg/dL)	2.0	-	2.2	2.1	1.4	2.6	3.2	-	-	-	< 0.9

**Figure 1:**
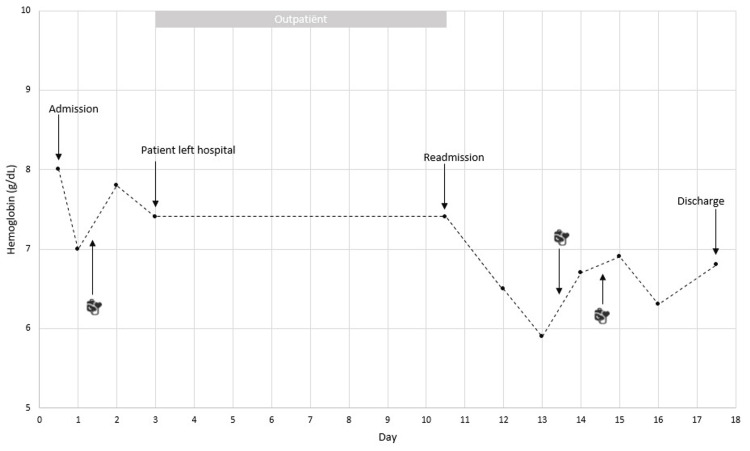
Trend of hemoglobin concentration during admissions

The patient received vigorous IV fluid hydration and opioid-based pain control as per VOC management protocol. Empiric antibiotic treatment (amoxicillin/clavulanic acid; one gram every six hours) was initiated for a presumed respiratory infection. On the second day of hospitalization, his Hb level dropped from 8.0 g/dL on admission to 7.0 g/dL for which he received one unit of antigen-matched (for the following clinically significant antigens: C- E- K- Fy^a^- S-) and crossmatch-compatible O negative packed red blood cells (pRBC) (Figure 1). No adverse events were reported following the transfusion and his Hb raised to 7.8 g/dL. On day four, the patient left the hospital against medical advice: despite the resolution of his VOC symptoms (he was pain-free and no longer required analgesia), his persistent respiratory symptoms along with the observed increase in C-reactive protein necessitated continued in-hospital monitoring.

Ten days later the patient was readmitted to our hospital with generalized body pain, most pronounced in both knees and in his left shoulder. Laboratory examination again showed normocytic normochromic anemia (Hb 7.4 g/dL) and acute hemolysis (Table 1), consistent with a further VOC. Inflammatory parameters had significantly improved compared to the previous hospitalization and he no longer experienced respiratory symptoms. The VOC management protocol was again initiated and the patient’s symptoms gradually began to improve. However, on day three of this hospitalization, the patient’s Hb level dropped to 6.5 g/dL (Figure 1) and two units of pRBCs were requested. No compatible units were found on low ionic strength saline (LISS)/anti-human globulin (AHG) phase crossmatch by column agglutination technique. Further workup showed a positive antibody screen (indirect antiglobulin test; IAT) and subsequent antibody identification revealed pan-reactivity in LISS-IAT phase testing at 37°C as well as in enzyme phase testing at 37°C with all test cells, except Fy(a-b-) cells. The autologous control was weakly positive. The pattern was suggestive of the presence of the previously detected anti-E alloantibody together with at least one new alloantibody, most likely against the rhesus blood group system or a high-frequency antigen (HFA) of the Duffy blood group system. The poly-specific direct antiglobulin test (DAT) was weakly positive with weakly positive anti-IgG and negative anti-C3b/C3d. Analysis of the eluate prepared from the patient’s red cells showed several weak reactions without demonstrable specificity.

Given the suspicion of the presence of one or more - yet to be identified and possibly rare - alloantibodies, and in the search for crossmatch-compatible pRBCs, patient samples were sent to the Belgian Red Cross-Flanders reference laboratory for additional testing. Two pheno-identical and crossmatch-compatible Fy(a-b-) units were identified in the frozen blood bank of the Belgian Red Cross-Flanders, but neither of these could be obtained rapidly. Therefore, the decision was made to manage the patient overnight without transfusion and to reevaluate the need for transfusion the next morning. The following morning, his Hb had dropped further to 5.9 g/dL and transfusion of the two compatible units was indicated. As a precaution, the patient was transferred to the ICU for continuous hemodynamic monitoring, high-dose IV corticosteroids were given prior to transfusion and each unit was administered by slow infusion over four hours. No adverse events were reported during or after transfusion and the patient’s Hb improved moderately to 6.9 g/dL (Figure 1).

Simultaneously, additional testing was performed at the reference center. Crossmatching with some RBCs with a rare phenotype (i) RhNull (D-C-c-E-e-), Fy(a+b-); ii) D-,- (D+C-c-E-e-), Fy(a+b+); and iii) HrB- (RH:-34), Fy(a-b-)) pointed to the presence of anti-Fy3 antibodies. In addition, antibody identification after alloabsorption revealed that the patient’s serum also contained anti‑E and anti‑Jk^b^ antibodies. Pheno- and genotyping of his RBCs was consistent with anti-E and anti-Jk^b^ alloimmunization, however for anti-Fy3 there was a discrepancy between the historical genotyping results (Fy^a^-Fy^b^+) and the current RBC serological antigen typing (Fy^a^-Fy^b^-). To further elaborate on this discrepancy, extended molecular typing for a point mutation in the GATA-1 binding motif of the erythroid promotor region of the FY*B gene (FY*B^ES^) and other rare silencing single-nucleotide polymorphisms (SNPs) was performed [7,8]. This had not been done during the initial molecular testing in 2012. FY*B^ES^ was found to be present homozygously.

Based on the additional immunohematological tests, the advice was formulated that the patient should receive C- E- K- Fy^a^- Fy^b^- Jk^b^- and S-negative blood. A subsequent, new nationwide search for compatible units yielded only two additional frozen units of fully phenotype-compatible pRBCs. Therefore, given the improved Hb level and the patient’s hemodynamic stability, further transfusion was avoided unless his Hb level would drop below 6 g/dL (Figure 1). Fortunately, the patient recovered without further need for transfusion and he was discharged eight days later.

## Discussion

Fy3 is an HFA that belongs to the Duffy blood group system and resides on the Duffy glycoprotein or DARC. Anti-Fy3 alloantibodies are rare, though clinically significant as they have been associated with hemolytic disease of the fetus and newborn as well as with both acute and delayed hemolytic transfusion reactions [9]. These antibodies solely arise in individuals with a Duffy null phenotype (Fy_null_; Fy(a-b-)), which is very rare in most ethnic populations (<1%), but prevalent in 68% of Africans and people of African descent [7-9]. This ethnic variation stems from the Fy_null_ phenotype protective advantage for populations living in malaria-endemic areas, as Fy loss protects against malaria invasion by *Plasmodium vivax* [9,10]. In a minority of cases, the Fy_null_ phenotype is the result of a frameshift or nonsense mutation that introduces a premature stop codon causing DARC to be absent from all tissues. This type of mutation occurs predominantly in Caucasian and Asian Fy_null_ individuals [7,8]. More commonly, the Fy_null_ phenotype is the result of a point mutation (c.-67T>C) in the GATA-1-binding motif of the erythroid promotor region of the *FY* gene that abolishes Fy^b^ expression on RBCs, while the glycoprotein is expressed normally on other cell types. This erythroid-specific mutation is found predominantly in Africans and people of African descent [7,11]. Individuals with the promotor mutation are not predisposed to developing anti-Fy^b^, but may occasionally develop anti-Fy3 despite the expression of Fy^b^ on their nonerythroid tissues [8]. In our case, the Fy_null_ phenotype and the emergence of anti-Fy3 could be attributed to the presence of the erythroid-specific GATA-1-binding motif mutation. However, unlike most cases, the anti-Fy3 alloantibodies were here unaccompanied by concurrent or existing anti-Fy^a^ alloimmunization, rendering this an atypical case of anti-Fy3 development [12-17].

Current guidelines on RBC transfusion in SCD recommend prophylactic serological phenotyping or molecular genotyping to support extended antigen matching between blood donors and patients, and to lower the prevalence of alloimmunization [4,9,18]. Therefore, the patient’s red cell antigen profile had been obtained in the past by molecular genotyping. However, at that time, no molecular analysis of the GATA-1-binding motif was carried out, which at present resulted in the observation of a discrepancy between RBC pheno- and genotype due to the absence of *FY* gene expression in the erythroid lineage [19]. As such, extended RBC genotyping was here of added value in resolving this discrepancy and in identifying the newly formed alloantibodies. Nevertheless, this information would not have affected the prior selection of transfusable units for this patient, since Fy(a-b+) units can be used for transfusions in individuals homozygous for FY*B^ES^. Only in Fy_null_ individuals who have already developed anti-Fy^a^, the use of Fy(a−b−) units is recommended to avoid additional alloimmunization with anti-Fy3 (and anti-Fy^b^) [4,5].

A look back at the phenotype of the pRBC unit transfused during the initial hospitalization showed that this unit was Fy(a-b+) and Jk(a+b+), which underscores that the new anti-Jk^b^ alloantibody emerged through exposure to Jk^b^ antigen. However, the reason why the patient developed anti-Fy3 alloantibodies during this transfusion rather than during a previous transfusion with a Fy(a-b+) unit is unclear.

The poor representation of Africans or individuals of African descent in the Belgian donor pool (predominantly consisting of individuals of European descent in whom the prevalence of Fy^b^ antigen is high) greatly complicates the search for compatible pRBC units for an SCD patient in whom the presence of anti-Fy3 alloantibodies restricts compatibility to individuals of African ethnicity [20]. Furthermore, the presence of other clinically significant alloantibodies (anti-E and anti-Jk^b^) adds another layer of complexity to this patient’s transfusion management. This complexity is reflected by the limited number of phenotype-compatible units available in Belgium for our patient and highlights the difficulty of ensuring timely transfusion in SCD patients with such complex alloimmunization profiles. Besides, it emphasizes the importance of maintaining - at least nationwide - a diverse and adequately stocked blood inventory to meet the unique transfusion needs of such patients with complex alloimmunization profiles. Fortunately, our patient didn’t require urgent or extensive red cell transfusion during his second admission, otherwise difficult decisions would have had to be made.

## Conclusions

In summary, Fy3 is a high-frequency antigen and antibodies against Fy3 only arise in individuals with the rare Fy_null_ phenotype. Red cell transfusion in individuals with anti-Fy3 alloimmunization requires the selection of Fy(a-b-) pRBC units, which are scarce in European countries. Furthermore, if the patient also has other clinically significant antibodies (as is often the case with sickle cell patients who have undergone multiple transfusion), the likelihood of finding crossmatch-compatible pRBC units is even further reduced. The case presented here illustrates the complexity of transfusion in SCD patients, specifically focusing on challenges encountered in identifying rare anti-Fy3 alloantibodies and finding compatible pRBC units for transfusion. The case also emphasizes the importance of comprehensive immunohematological testing and the value of collaborative transfusion strategies in facilitating timely transfusions for SCD patients with rare alloimmunization profiles.
